# Evaluating the Optimal Approach: Effectiveness of Medical vs. Surgical Treatment for Primary Bladder Neck Obstruction in Young Males

**DOI:** 10.7759/cureus.81072

**Published:** 2025-03-24

**Authors:** Ankur Mittal, Avin Singhal, Vikas Panwar, Deelip Kumar Singh, Siddharta Saxena, Mehul Agarwal, Nalin Srivastava

**Affiliations:** 1 Urology, All India Institute of Medical Sciences, Rishikesh, Rishikesh, IND

**Keywords:** lower urinary tract symptoms, medical, primary bladder neck obstruction, surgical, urodynamics, young males

## Abstract

Background

Primary bladder neck obstruction (PBNO) is a functional obstruction at the bladder neck causing lower urinary tract symptoms (LUTS) in young males. Treatment options include medical therapy with alpha-blockers and surgical intervention, such as bladder neck incision (BNI). This study compares the clinical and urodynamic outcomes of these modalities.

Methodology

A retrospective analysis was conducted on 67 males (<40 years) with PBNO diagnosed via video urodynamics. Patients were divided into medical (*n* = 39) and surgical (*n *= 28) groups. Symptom scores (International Prostate Symptom Score [IPSS], Urogenital Distress Inventory-6 [UDI-6]) and urodynamic parameters (Qmax, Qavg, post-void residual [PVR]) were assessed at baseline, two, four, and six weeks, and six months. Statistical analysis evaluated changes between groups.

Results

Both groups showed significant improvements, but the surgical group demonstrated superior outcomes. By six months, the surgical group had a greater reduction in IPSS (11.9 ± 4.40 vs. 4.38 ± 3.73; *P *< 0.001) and UDI-6 scores. Qmax increased significantly more in the surgical group (+5.59 ± 4.08 mL/s vs. +2.54 ± 3.85 mL/s; *P *< 0.001).

Conclusions

Surgical intervention provides greater and more sustained symptom relief and urodynamic improvement compared to medical therapy. While alpha-blockers are effective for mild cases, early surgical treatment should be considered in severe obstruction to prevent complications and ensure better outcomes.

## Introduction

Primary bladder neck obstruction (PBNO) is an infrequent yet notable cause of lower urinary tract symptoms (LUTS) in young males [1]. In contrast to older men, where benign prostatic hyperplasia (BPH) is the primary cause of urinary obstruction, PBNO in younger males is defined by functional obstruction at the bladder neck without significant prostatic enlargement. This condition presents with various distressing symptoms, such as difficulty initiating urination, diminished urinary stream, sensation of incomplete bladder evacuation, increased urinary frequency, and nocturia [2].

It is often necessary to do specialized urodynamic tests, especially video urodynamic studies (vUDS) [3], to diagnose PBNO when there are no anatomical problems, like urethral strictures or calculi. vUDS can help identify abnormalities in the bladder and urethral function that may be contributing to the symptoms of PBNO. Additionally, treatment options for PBNO may include medications, minimally invasive procedures, or surgery depending on the severity of the condition. The prevalence of PBNO is likely underreported, as diagnostic delays are common [4]. Misdiagnosis often leads to unnecessary antibiotic treatments and delays in appropriate management, potentially worsening the symptoms and quality of life for individuals with PBNO.

Urodynamic studies play a pivotal role in differentiating PBNO from other causes of urinary dysfunction, guiding clinicians in the appropriate management of this condition. These studies validate the existence of elevated bladder pressures, diminished urine flow, and constriction of the bladder neck during micturition. Untreated PBNO may lead to severe complications such as bladder wall hypertrophy, chronic urinary retention, and obstructive uropathy, emphasizing the critical need for timely recognition and management.

For patients with PBNO, medical therapy with alpha-blockers aims to alleviate symptoms by relaxing the bladder neck muscles, while surgical intervention, such as bladder neck incision (BNI), focuses on widening the obstructed area to improve urinary flow [5,6]. Despite the demonstrated efficacy of medical therapy and surgical intervention for PBNO, there is a notable lack of comprehensive studies comparing their long-term effectiveness, indicating a crucial need for further research in this area. In this study, the effects of alpha-blockers and surgery, specifically a BNI, on treating PBNO will be looked at. The main focus will be on clinical, urodynamic, and patient-reported outcomes over a six-month evaluation period.

## Materials and methods

Study design and patient selection

This retrospective observational study was conducted to evaluate the clinical and urodynamic outcomes of young males (under 40 years of age) diagnosed with PBNO based on vUDS. The study included patients who received treatment between January 2022 and November 2024 at our institute.

A total of 67 participants met the inclusion criteria and were followed for six months post-treatment. Patients were divided into two groups based on their treatment modality:

Medical Management Group (*n *= 39): Patients receiving alpha-blocker therapy.

Surgical Management Group (*n *= 28): Patients undergoing bladder neck incision (BNI) or transurethral incision of the prostate (TUIP).

The selection of treatment was personalized, based on the severity of symptoms, urodynamic findings, patient preference, and physician recommendations. The aim was to optimize therapeutic outcomes for each patient.

Males under the age of 40 years with a diagnosis of PBNO confirmed by vUDS, who received either medical or surgical treatment and were available for a six-month follow-up, were included in the study.

Patients were excluded if they had a history of previous bladder neck or prostate surgery, the presence of urethral stricture, bladder or urethral calculi, or neurogenic bladder dysfunction, or if they required urgent surgical intervention at presentation due to hydronephrosis or obstructive uropathy

Data collection and outcome measures

Patient medical records were meticulously reviewed to collect demographic details, symptom scores, and urodynamic parameters at baseline and follow-up intervals (two, four, and six weeks and six months).

The primary clinical outcome measures included the International Prostate Symptom Score (IPSS) for assessing symptom severity and the Urogenital Distress Inventory-6 (UDI-6) for evaluating urinary discomfort and distress.

The urodynamic outcome measures included maximum urine flow rate (Qmax) (mL/s), average urine flow rate (Qavg) (mL/s), and post-void residual volume (PVR) (mL).

Statistical analysis

All statistical analyses were conducted using SPSS Version 20 (IBM Corp., Armonk, NY).

Continuous variables (e.g., Qmax, Qavg, PVR, IPSS, UDI-6 scores) were reported as mean ± standard deviation (SD).

Categorical variables (e.g., treatment modality, symptom improvement) were analyzed using the Chi-square test.

Within-group and between-group comparisons of clinical and urodynamic outcomes at different time points were assessed using paired and independent t-tests, respectively.

A *P*-value < 0.05 was considered statistically significant.

## Results

Demographic attributes

The mean age in the medical group was 17.0 ± 5.48 years, whereas in the surgical group, it was 19.11 ± 3.85 years. The difference was not statistically significant (*P* = 0.68), suggesting a comparable age distribution between the two groups.

Enhancement in IPSS scores

**Figure 1:**
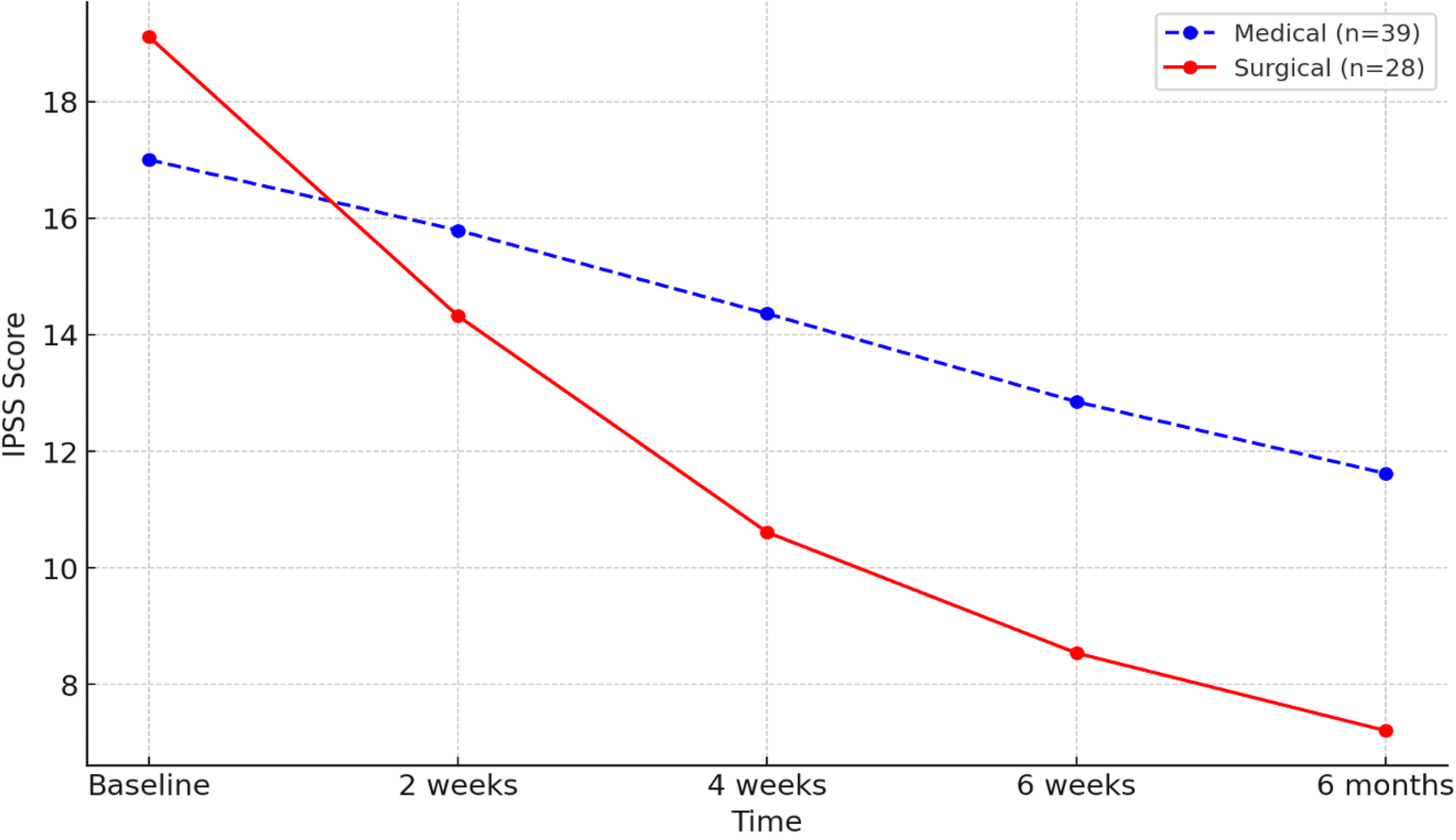
IPSS comparison between medical vs. surgical. IPSS, International Prostate Symptom Score

The research evaluated variations in the IPSS over time in patients undergoing medical and surgical treatment for PBNO (Figure 1, Table 1).

**Table 1:** Comparison of IPSS score between both groups at different intervals. IPSS, International Prostate Symptom Score

IPSS score	Medical (*n* = 39)	Surgical (*n* = 28)	*P-*value
Baseline	17.0 ± 5.48	19.11 ± 3.85	0.19
2 weeks	15.79 ± 5.20	14.32 ± 3.96	0.15
4 weeks	14.36 ± 5.30	10.61 ± 4.68	0.01
6 weeks	12.85 ± 5.31	8.54 ± 4.22	<0.01
6 months	11.62 ± 4.79	7.21 ± 3.47	<0.001
Change in IPSS	5.38 ± 3.73	11.89 ± 4.40	<0.001

Baseline Scores

At baseline, the average IPSS was 17.0 ± 5.48 in the medical cohort and 19.11 ± 3.85 in the surgical cohort. There was no statistically significant difference (*P* = 0.19).

Two Weeks

At the two-week follow-up, both groups exhibited enhancements in IPSS. The average score declined to 15.79 ± 5.20 in the medical group and 14.32 ± 3.96 in the surgical group, with no statistically significant difference observed between the groups (*P* = 0.15).

Four Weeks

The surgical group exhibited a significantly greater improvement, with a mean score of 10.61 ± 4.68, compared to 14.36 ± 5.30 in the medical group (*P* = 0.01).

Six Weeks

By six weeks, the surgical group continued to show superior outcomes, with a mean score of 8.54 ± 4.22 compared to 12.85 ± 5.31 in the medical group (*P* < 0.01).

Six Months

The surgical group exhibited significantly superior outcomes, attaining a mean score of 7.21 ± 3.47, in contrast to 12.62 ± 4.79 in the medical group (*P* < 0.001).

Comparative analysis of UDI scores

**Figure 2:**
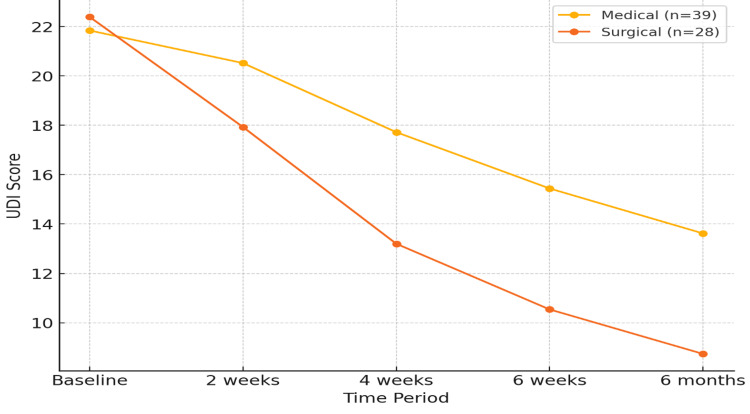
Comparison of UDI-6 scores between medical and surgical management. UDI-6, Urogenital Distress Inventory-6

The UDI-6 scores were analyzed between the medical and surgical cohorts at various time intervals to assess the amelioration of symptoms associated with PBNO (Figure 2, Table 2).

**Table 2:** Comparison of UDI score between both groups at different intervals. UDI, Urogenital Distress Inventory

UDI score	Medical (*n* = 39)	Surgical (*n* = 28)	*P*-value
Baseline	21.84 ± 8.39	22.39 ± 4.64	0.51
2 weeks	20.52 ± 7.66	17.93 ± 5.15	0.10
4 weeks	17.72 ± 7.83	13.20 ± 4.54	<0.01
6 weeks	15.44 ± 7.21	10.54 ± 3.87	<0.001
6 months	13.62 ± 7.18	8.74 ± 5.24	<0.01
Change in UDI at 6 m	8.22 ± 5.28	13.64 ± 5.93	0.001

Baseline Scores

At baseline, the average UDI score was 21.84 ± 8.39 in the medical cohort and 22.39 ± 4.64 in the surgical cohort. The disparity was not statistically significant (*P* = 0.51).

Two Weeks

By the two-week follow-up, both groups showed improvement. The mean UDI score decreased to 20.52 ± 7.66 in the medical group and 17.93 ± 5.15 in the surgical group. However, the difference was not statistically significant (*P* = 0.10).

Four Weeks

At four weeks, the surgical group demonstrated significantly greater symptom improvement, with a mean UDI score of 13.20 ± 4.54 compared to 17.72 ± 7.83 in the medical group (*P* < 0.01).

Six Weeks

By six weeks, the surgical group maintained superior outcomes, achieving a mean score of 10.54 ± 3.87 compared to 15.44 ± 7.21 in the medical group (*P* < 0.001).

Six Months

At the six-month follow-up, the surgical group continued to exhibit significantly better results, with a mean score of 8.74 ± 5.24 compared to 14.92 ± 7.18 in the medical group (*P* < 0.01).

Uroflowmetry results: Qmax, Qavg, and voided volume (VV)

The improvement in maximum urine flow rate (Qmax) and Qavg further demonstrated the superiority of surgical intervention (Table 3).

**Table 3:** Comparison of different parameters between both groups at baseline and six months.

	Medical (*n* = 39)	Surgical (*n* = 28)	*P*-value
Qmax	12.90 ± 3.89	11.92 ± 4.84	0.32
Qmax (six months)	15.44 ± 3.85	17.51 ± 5.24	0.07
Qavg	6.04 ± 2.57	5.66 ± 4.01	0.19
Qavg (six months)	7.95 ± 2.63	9.18 ± 2.63	0.03

The surgical cohort exhibited a more significant enhancement in Qmax, rising from 11.92 ± 4.84 mL/s to 17.51 ± 5.24 mL/s over a period of six months, in contrast to the medical cohort, which increased from 12.90 ± 3.89 to 15.44 ± 3.85 mL/s.

The *P*-value for Qmax alteration was <0.001.

The average flow rate (Qavg) exhibited greater improvement in the surgical group (3.52 ± 4.03 mL/s) than in the medical group (1.90 ± 2.67 mL/s) (*P* = 0.02).

VV in both groups showed a marginal increase; however, the difference was not statistically significant (*P* = 0.37).

## Discussion

PBNO refers to a functional obstruction at the bladder neck [7,8], typically seen in younger patients, often without structural abnormalities like prostate enlargement. It presents with symptoms of voiding difficulty, including weak stream, hesitancy, incomplete bladder emptying, and sometimes storage symptoms like frequency and urgency​. The primary mechanism involves failure of the bladder neck to relax during voiding, resulting in high detrusor pressures and incomplete emptying. Diagnosing PBNO is challenging, as it necessitates vUDS to distinguish it from other etiologies of LUTS, including urethral strictures or neurogenic bladder dysfunction [9].

Young males with PBNO frequently receive misdiagnoses of chronic prostatitis or are inappropriately treated with antibiotics due to symptom overlap. Consequently, there is frequently a considerable delay in diagnosis, resulting in extended discomfort and, in certain instances, advancing bladder dysfunction. Research, including that of Yang et al. [10], has underscored the necessity of early diagnosis via vUDS to prevent chronic bladder alterations and improve treatment efficacy.

This study sought to evaluate the effectiveness of medical versus surgical management in young males with PBNO. The findings indicate that although both methods enhance clinical and urodynamic results, surgical intervention provides quicker and more enduring advantages, especially in alleviating symptoms and restoring function. This discourse will elucidate the study's findings in relation to existing literature and examine the implications of the results.

Clinical and symptomatic outcomes: IPSS and UDI-6 scores

The study showed that both the IPSS and the UDI-6 scores got a lot better in patients who were treated with medicine or surgery. However, patients in the surgical group experienced markedly better outcomes. By six months, the surgical group had a mean reduction in IPSS of 11.9 ± 4.40 points, compared to 4.38 ± 3.73 points in the medical group (*P* < 0.001).

These findings align with prior research by Mittal et al. [11], which highlighted the long-term benefits of surgical procedures like BNI in managing PBNO. Alpha-blockers such as tamsulosin relax the bladder neck muscles, providing short-term symptom relief [12]. However, their effectiveness often plateaus because they do not address the underlying structural obstruction. According to Sudrania et al. [13], patients who have significant bladder outlet obstruction (e.g., Bladder Outlet Obstruction Index [BOOI] > 60) typically respond poorly to monotherapy with alpha-blockers. Surgical interventions effectively rectify the mechanical obstruction, leading to prolonged symptom relief.

Urodynamic outcomes

The surgical group demonstrated superior enhancements in urodynamic metrics, especially in maximum urine flow rate (Qmax). In six months, Qmax rose by 5.58 ± 7.09 mL/s in the surgical cohort, in contrast to 2.54 ± 3.41 mL/s in the medical cohort (*P* < 0.001). This enhancement underscores the capacity of surgical procedures to more efficiently alleviate the obstruction.

In agreement with our results, Sudrania et al. (2018) reported significant increases in Qmax after surgery in patients who were not responding to drug treatment. The superior results in the surgical cohort highlight the benefit of directly resolving the physical obstruction at the bladder neck.

Constraints of medical management

Alpha-blockers, such as tamsulosin, doxazosin, and alfuzosin, continue to be a prevalent first-line therapy for PBNO. These medications enhance urinary flow and alleviate symptoms by relaxing the smooth muscles of the bladder neck [14]. Although effective for mild to moderate cases, their advantages are frequently limited in severe obstruction and may decline over time.

While alpha-blockers offer a non-invasive alternative, they are not devoid of disadvantages. Adverse effects, including dizziness, orthostatic hypotension, and abnormal ejaculation, may affect adherence, especially in younger patients. In cases of significant obstruction or insufficient symptom management, extended medical treatment may postpone more conclusive interventions, heightening the risk of complications like bladder wall hypertrophy or urinary retention [15].

Surgical interventions such as BNI provide a highly efficacious remedy for patients with PBNO unresponsive to pharmacological treatment. BNI works by relieving the physical obstruction at the bladder neck, leading to improved urinary flow and symptom resolution. Research indicates that BNI yields better results than medical therapy, particularly in enhancing Qmax, reducing PVR, and improving quality of life.

Surgical interventions entail specific risks. Retrograde ejaculation, which refers to the backward flow of semen into the bladder during ejaculation, is a common side effect that can affect fertility, particularly in younger patients. Additional potential complications encompass urinary tract infections and, in infrequent instances, symptom recurrence necessitating repeat surgery. Innovations in surgical methodologies, such as laser-assisted interventions, have diminished complication rates and enhanced patient satisfaction. Thorough patient counseling regarding the risks and benefits is crucial for informed decision-making.

Treatment decisions for PBNO must be customized according to each patient's symptoms, clinical characteristics, and preferences. Alpha-blockers may serve as a suitable initial intervention for individuals exhibiting mild symptoms. Surgical intervention should be promptly considered for patients exhibiting severe obstruction or refractory symptoms [16]. Yang et al.'s research emphasizes the need for quick surgical intervention for patients with elevated obstruction indices (such as BOOI > 60) to prevent complications and improve long-term results [10]. 

Collaborative decision-making is essential to enhance treatment results. Factors including patient age, lifestyle, fertility considerations, and tolerance for possible side effects should inform the selection of therapy. Patients receiving medical management should have regular follow-ups to assess symptom progression and identify complications promptly. Proactive detection of treatment failure can avert severe consequences, including obstructive uropathy or hydronephrosis [17].

By weighing the risks and advantages of each treatment modality, clinicians can deliver personalized care that effectively meets the needs of patients with PBNO.

This study focuses on a significant yet under-researched population; however, it also has limitations. First, because clinical and patient factors are likely to have influenced treatment choice, the retrospective design introduces the possibility of selection bias. Second, the follow-up period of six months, while sufficient to capture early outcomes, may not reflect the long-term sustainability of symptom relief, particularly for medical therapy. Subsequent research with extended follow-up periods and randomized controlled methodologies is essential to corroborate these findings and investigate the long-term advantages and disadvantages of both treatment modalities.

This study does not compare various alpha blockers nor does it evaluate different types of surgical interventions performed.

Furthermore, innovations in minimally invasive surgical methods, including laser BNI, may diminish the likelihood of complications and enhance patient satisfaction.

## Conclusions

This study emphasizes the disparities in outcomes between medical and surgical interventions for PBNO in young males. Both methodologies resulted in enhancements in clinical symptoms and urodynamic metrics; however, surgical intervention exhibited markedly superior outcomes. During the six-month follow-up, patients who had surgery, like a BNI, had more significant drops in symptom scores (IPSS and UDI-6) and noticeable improvements in urodynamic metrics, such as Qmax. 

Alpha-blockers serve as an effective first-line treatment for patients with mild symptoms or those desiring non-invasive options; however, their efficacy declines in instances of severe obstruction or chronic symptoms. Surgical intervention, despite potential risks like retrograde ejaculation, provides a more conclusive solution by rectifying the mechanical obstruction at the bladder neck. These findings underscore the significance of personalized treatment strategies tailored to symptom severity, patient preferences, and clinical presentation. Prompt identification of treatment failure and timely surgical intervention can avert long-term complications, enhance quality of life, and offer enduring symptom relief for patients with PBNO.
